# Esophagopericardial fistula secondary to battery acid ingestion in a 3-year-old boy: a case report

**DOI:** 10.1186/s12245-026-01332-9

**Published:** 2026-07-31

**Authors:** Yasser Abdurabo Obadiel, Mohammed Al-Shehari, Aref Al-Hashedi, Mansour Al-Hameli, Nardeen Alqousi, Anas Al-Kubati, Abdullah Al-Kubati, Bilal Sleiay, Osama Al-Khawlani, Majeed Shallan

**Affiliations:** 1 Department of Surgery, Faculty of Medicine and Health Sciences, Sana’a University, Sana’a, Yemen; 2 Faculty of Medicine, 21 September University for Medical and Applied Sciences, Sana’a, Yemen; 3 Department of Radiology, Faculty of Medicine and Health Sciences, Sana’a University, Sana’a, Yemen; 4 Department of Surgery, Al-Thawra Modern General Hospital, Sana’a, Yemen; 5 Faculty of Medicine, Hama University, Hama, Syrian Arab Republic; 6 Faculty of Medicine, Sana’a University, Sana’a, Yemen; 7 Department of Anesthesiology and Intensive Care, 21 September University for Medical and Applied Sciences, Sana’a, Yemen

**Keywords:** Esophagopericardial fistula, Corrosive ingestion, Battery acid, Pediatric surgery, Sepsis, Esophagectomy

## Abstract

**Background:**

Esophagopericardial fistula (EPF) is an extremely rare and life-threatening condition associated with a high mortality rate, particularly when diagnosis and intervention are delayed. Here we present the case of a 3-year-old boy who developed EPF secondary to accidental battery acid ingestion.

**Case presentation:**

A 3-year-old Yemeni boy presented with persistent epigastric pain, fever, and progressive dyspnea three weeks after accidental ingestion of battery acid, having initially received conservative supportive management at a regional hospital before delayed referral. Clinical examination revealed tachycardia, tachypnea, and a pericardial friction rub. Laboratory investigations demonstrated leukocytosis, severe thrombocytopenia, anemia, hypoalbuminemia, and markedly elevated inflammatory markers, consistent with sepsis. Imaging studies, including an iso-osmolar contrast swallow and CT, identified a 12-mm esophagopericardial fistula located in the distal esophagus, 2.5 cm above the gastroesophageal junction, accompanied by pyopneumopericardium. Following initial stabilization, the patient underwent a right posterolateral thoracotomy. Intraoperatively, gross inspection revealed extensive full-thickness ischemic necrosis and friability of the distal esophagus with inflammation extending to the pericardium, necessitating radical esophagectomy. The pericardium was opened, thoroughly irrigated with warm sterile saline, and a wide pericardial window was created for continuous drainage. An esophagectomy with gastric pull-up reconstruction was performed to achieve source control. Despite aggressive postoperative critical care, the patient developed refractory sepsis with multiorgan failure and died on the sixth postoperative day.

**Conclusion:**

This case highlights the poor prognosis of esophagopericardial fistula following corrosive ingestion in children, especially with delayed presentation and subacute progression. Early recognition and immediate referral to specialized tertiary pediatric surgery centers are paramount.

## Background

Esophagopericardial fistula (EPF) is an extremely rare pathological communication between the esophagus and the pericardium. It represents a surgical emergency due to its high mortality, which has been reported to range between 50% and 80% [1]. EPF may result from esophageal malignancies, trauma, infections, iatrogenic injury, or corrosive substance ingestion [2].

Corrosive substances, especially strong acids, cause coagulative necrosis of the esophageal wall, resulting in progressive transmural injury. This damage may evolve silently or present with insidious, non-specific symptoms, leading to delayed perforation or fistula formation weeks after the initial exposure [3–5].

Patients typically present with nonspecific symptoms such as chest pain, dysphagia, fever, or dyspnea, which can mimic pneumonia or idiopathic pericarditis. This overlap frequently leads to diagnosis delays [6]. Diagnosis relies on a multimodal imaging strategy combining contrast-enhanced computed tomography (CT), esophagoscopy, and esophagography, supplemented by careful endoscopy when appropriate [7]. Management depends on the severity of the condition, with surgical intervention necessary in cases involving extensive mediastinal and pericardial contamination, persistent bleeding, hematoma accumulation, or abscess formation [6].

We present a rare and fatal case of corrosive-induced EPF in a 3-year-old boy following battery acid ingestion, emphasizing the diagnostic challenges, surgical decision-making, management limitations in a resource-constrained setting, and the poor prognosis associated with delayed presentation.

This work has been reported in line with the CARE criteria [8].

## Case presentation

A 3-year-old Yemeni boy presented to our tertiary center following accidental battery acid (sulfuric acid) ingestion.

On Day 0, the child was accidental ingestion exposed and was initially evaluated at a regional facility, where he received conservative supportive care and was subsequently discharged. Over Days 1–20, the patient did not experience a truly asymptomatic period; rather, his clinical course was characterized by persistent, underestimated symptoms—including epigastric pain exacerbated by oral intake, progressive dysphagia, low-grade fevers, night sweats, and coughing during feeds—reflecting progressive transmural esophageal necrosis and delayed eschar sloughing.

On Day 21 (Admission), he presented to our tertiary center in severe respiratory distress with a toxic appearance, fever (38.1 °C), tachycardia (105 bpm), tachypnea (36 bpm), and a distinct pericardial friction rub on cardiac auscultation. Initial laboratory investigations confirmed severe sepsis with marked leukocytosis (21 × 10³/µL), severe thrombocytopenia (38 × 10³/µL), anemia (hemoglobin 9.4 g/dL), hypoalbuminemia, and elevated C-reactive protein (48 mg/L). Blood cultures were drawn upon admission; however, both ultimately yielded no growth, likely masked by pre-procedural broad-spectrum antimicrobial administration.

Comprehensive diagnostic workup on Day 21—comprising echocardiography revealing a 7.2 mm pericardial effusion without overt features of cardiac tamponade, a contrast swallow study demonstrating contrast extravasation into the pericardium through a 12 mm fistula located 2.5 cm above the gastroesophageal junction, a chest CT confirming pyopneumopericardium with air-fluid levels, and an upper gastrointestinal endoscopy directly visualizing the distal defect and adjacent stricture—establishing the diagnosis of an esophagopericardial fistula.

On Day 22 (Operative Day), following initial resuscitation, the patient underwent an emergency right posterolateral thoracotomy featuring radical distal esophagectomy, formal pericardial opening with warm saline irrigation, intraoperative fluid sampling for microbiological culture (which ultimately yield no growth), creation of a wide pericardial window, and single-stage gastric pull-up reconstruction.

During Postoperative Days 1–5, despite initial intraoperative stability, the patient developed progressive septic shock with escalating vasopressor requirements, multiorgan dysfunction syndrome (MODS), and refractory hypotension, culminating in his death on Postoperative Day 6 secondary to refractory septic shock and multiorgan failure.

### Diagnostic workup sequence & considerations

On admission, initial stabilization included strict nil per os (NPO) status, intravenous proton pump inhibitors, aggressive fluid resuscitation, and broad-spectrum intravenous antibiotics with empirical antifungal coverage (fluconazole) to cover potential fungal colonizers commonly observed in lower gastrointestinal and esophageal perforations.

**Fig. 1:**
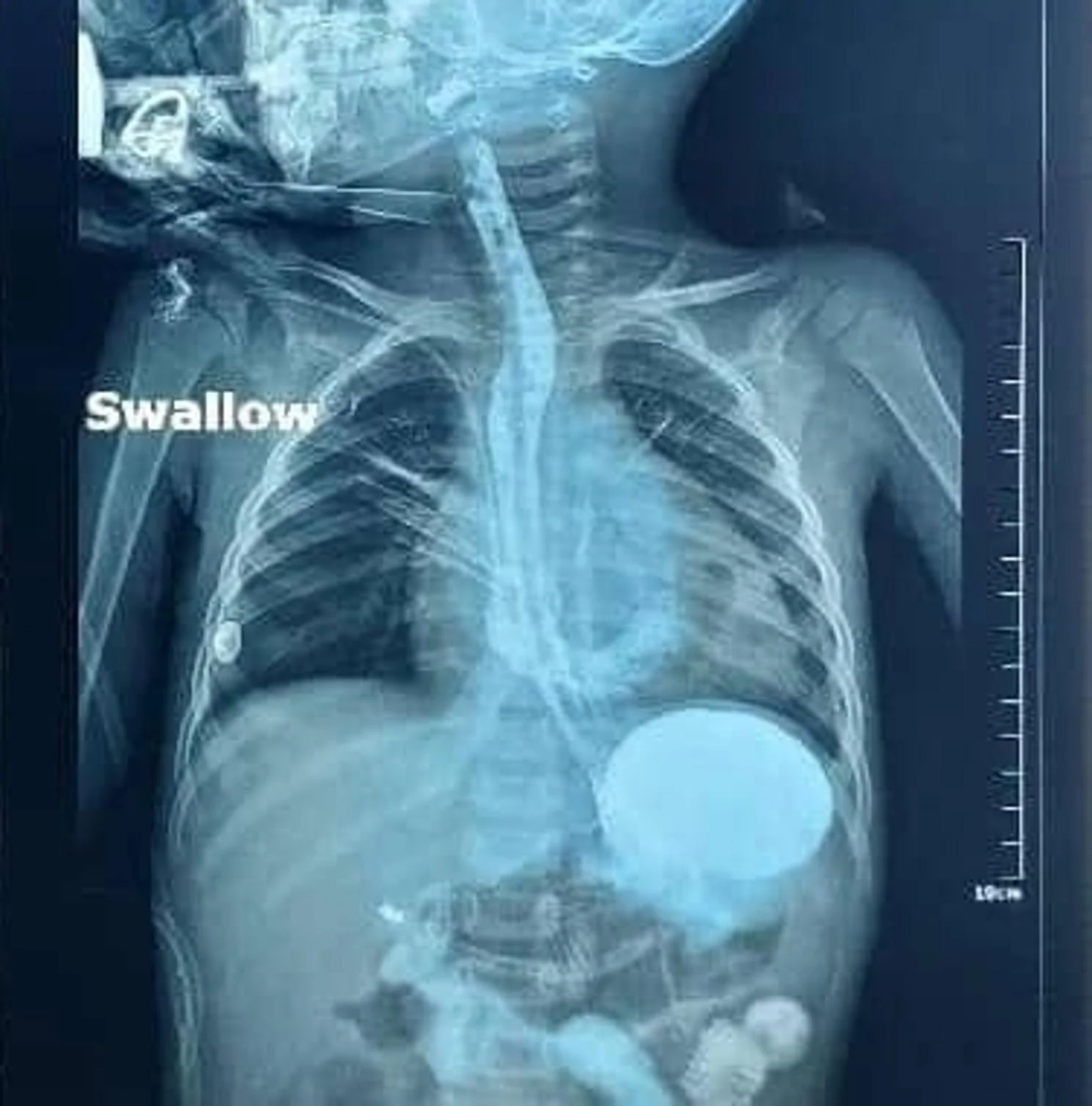
Preoperative water-soluble contrast esophagogram: An iso-osmolar contrast swallow study showing extravasation of contrast into the pericardium with mild distal esophageal stenosis

**Fig. 2:**
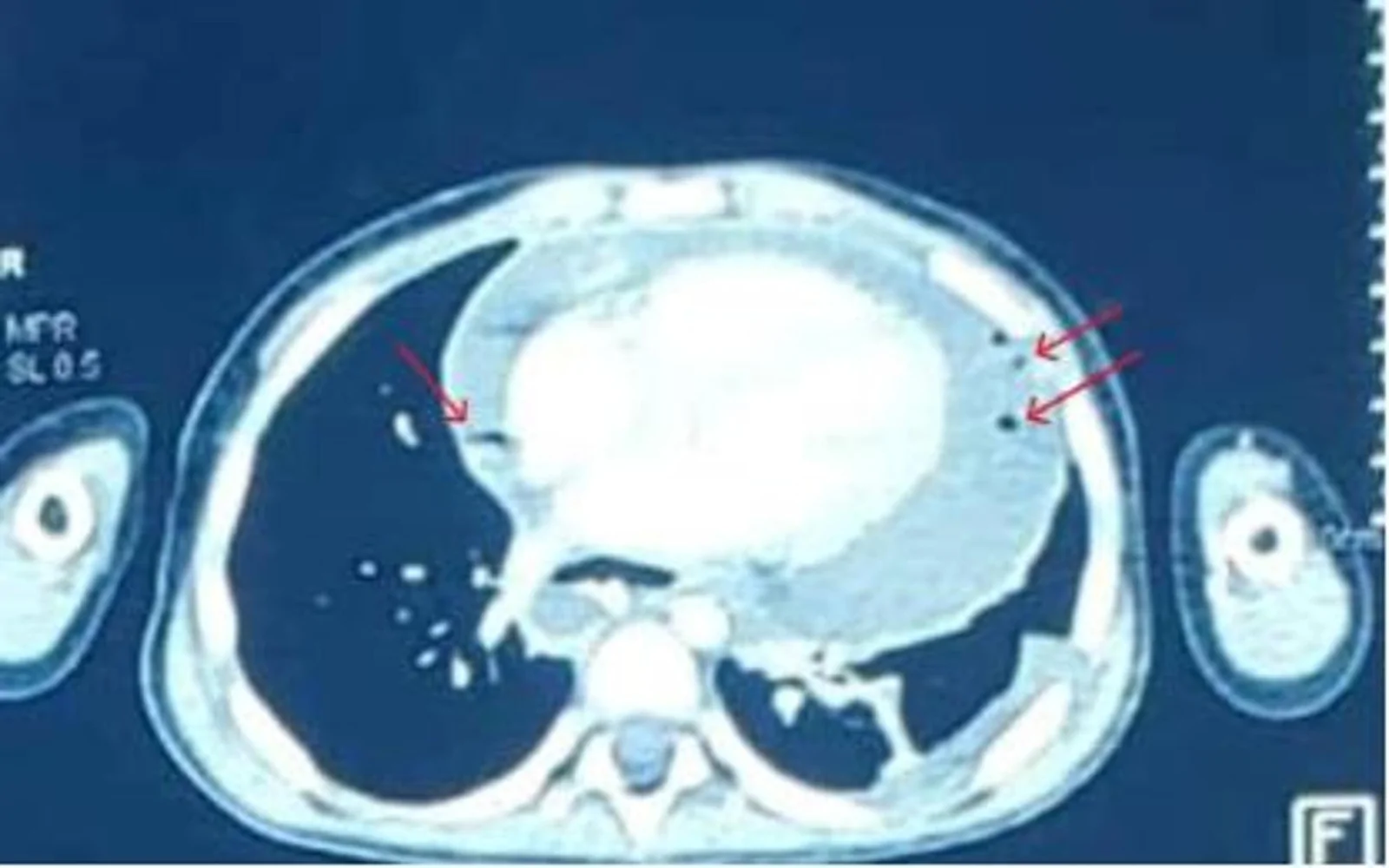
Axial contrast-enhanced CT of the chest showing circumferential pericardial effusion with pericardial thickening and enhancement. The red arrow points to multiple air bubbles (pneumopericardium) along with fluid accumulation, establishing the diagnosis of pyopneumopericardium secondary to distal esophagopericardial fistula

The diagnostic sequence was systematically executed to minimize procedural risks. Transthoracic echocardiography was performed first, confirming a circumferential pericardial effusion measuring 7.2 mm in thickness without hemodynamic evidence of cardiac tamponade (such as right ventricular collapse). An iso-osmolar water-soluble contrast swallow study was then conducted, demonstrating extravasation of contrast from the distal esophagus into the pericardial space through a 12-mm fistulous tract located 2.5 cm proximal to the gastroesophageal junction (Fig. 1). Contrast-enhanced chest CT confirmed the fistulous tract and demonstrated air-fluid levels within the pericardium, diagnostic of pyopneumopericardium (Fig. 2).

**Fig. 3:**
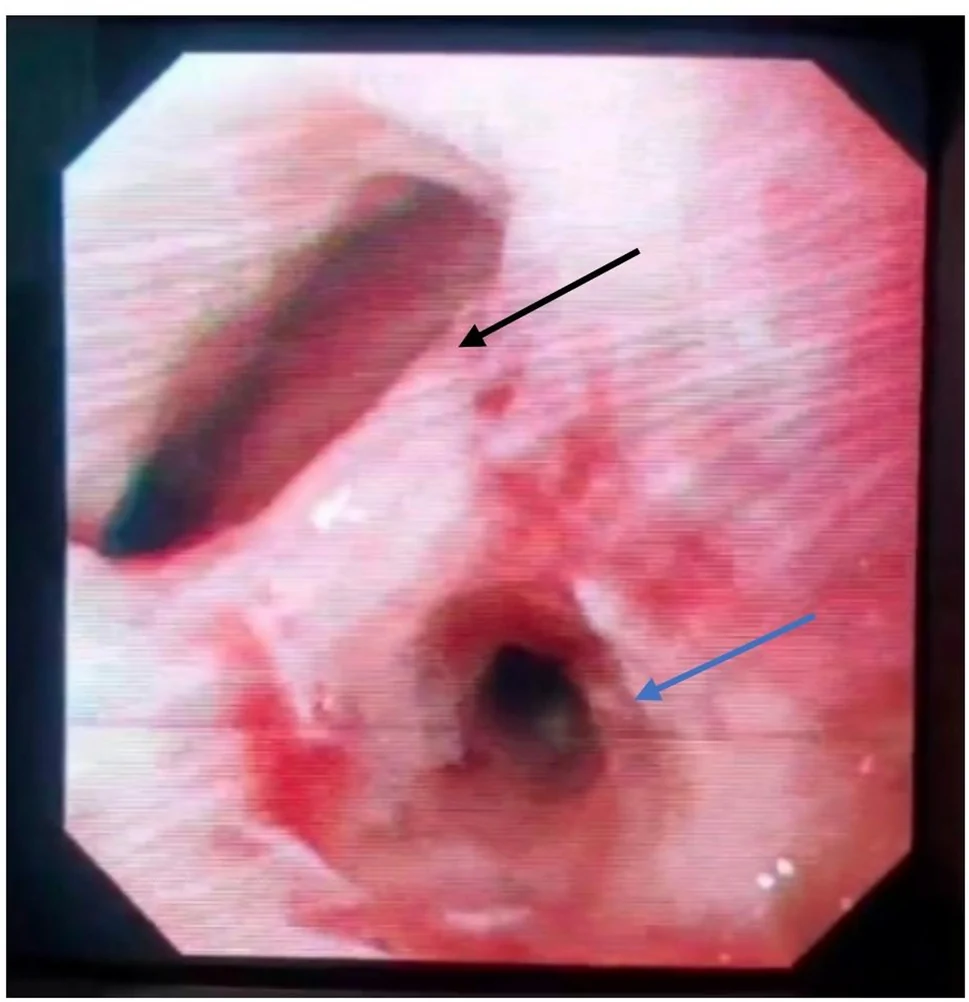
Upper gastrointestinal endoscopy visualization. Endoscopic view of the distal esophagus showing a large, transmural fistulous defect (black arrow) with surrounding inflamed mucosal edges, alongside mild distal esophageal luminal stenosis (blue arrow) proximal to the gastroesophageal junction

Upper gastrointestinal endoscopy (EGD) was intentionally performed last as a targeted diagnostic tool to directly map the fistula site, evaluate mucosal viability, and rule out coexisting gastric injury (Fig. 3). To avoid air-insufflation barotrauma, tension pyopneumopericardium, or systemic air embolism in the presence of a known pericardial communication, endoscopy was carried out under strict, low-pressure insufflation conditions.

### Operative management & pericardial handling

Given the patient’s deteriorating septic state, large fistula size (12 mm), and the complete unavailability of pediatric-sized advanced endoscopic devices—specifically pediatric fully covered self-expanding metal stents (FC-SEMS), endoscopic vacuum therapy (EVT), over-the-scope clips (OTSC), or endoscopic suturing systems in our resource-limited facility—definitive open surgical intervention was mandatory.

After multidisciplinary consultation, the patient underwent a right posterolateral thoracotomy. Intraoperative exploration revealed extensive full-thickness necrosis and friability of the distal esophageal tissue, which completely precluded any attempt at primary fistula repair or local tissue suturing. Addressing the pericardial space was a primary intraoperative objective: the pericardium was formally opened, inspected, and thoroughly debrided and irrigated with warm sterile saline. While no thick macroscopic purulent collection was encountered, inflammatory fluid and trapped gas were evacuated. Microbiological cultures of blood and intraoperative pericardial fluid were obtained; however, they yielded no growth, likely masked by pre-procedural broad-spectrum antimicrobial administration. To ensure continuous source control and prevent postoperative fluid re-accumulation or tamponade, a wide pericardial window was established, allowing continuous drainage into the right pleural cavity, where a dedicated chest drain was positioned.

The stomach was inspected and confirmed to be completely viable with robust vascular perfusion, having been spared from corrosive destruction. Facing transmural esophageal destruction, a radical esophagectomy was performed. Due to the emergency nature of the resection and primary focus on immediate source control, histopathological examination of the resected specimen was not performed; diagnosis of tissue non-viability was established entirely on direct gross intraoperative inspections.

The surgical choice between single-stage reconstruction and a staged damage-control approach (cervical esophagostomy, gastric closure, and feeding jejunostomy) was extensively debated. The multidisciplinary team decided upon an immediate gastric pull-up reconstruction based on six key intraoperative and contextual rationale. First, there was a favorable intraoperative environment where, despite the patient’s septic state, intraoperative exploration revealed a lack of gross macroscopic contamination (e.g., thick pus or food debris) within the mediastinal and pericardial spaces, with inflammatory fluid being strictly localized and easily manageable with irrigation. Second, the conduit quality was excellent, as the stomach was inspected and confirmed to be entirely viable with robust vascular perfusion, having been spared from the corrosive acid. Third, the patient demonstrated notable physiological reserve, maintaining hemodynamic stability throughout the esophagectomy, which prompted the team to opt for a definitive procedure to avoid the physiological “second hit” of a future major reconstruction. Fourth, the immediate reconstruction provided definitive source control through the complete removal of the necrotic esophagus and immediate restoration of gastrointestinal continuity. Fifth, geographical and post-operative care limitations weighed heavily; creating a temporary cervical esophagostomy (spit fistula) and feeding jejunostomy would have committed the child to complex, long-term stoma management, which, given the family’s residence in a remote rural area with poor access to specialized healthcare, posed a prohibitively high risk of outpatient morbidity—such as severe stoma-site excoriation, uncontrolled secretions, tube displacement, and subsequent malnutrition. Finally, regarding the nature of the outcome, the patient’s post-operative clinical course showed no evidence of an anastomotic leak or technical failure of the reconstruction, with mortality driven by the severe pre-existing systemic septic burden.

### Postoperative course

Postoperatively, despite initial intraoperative stability, the patient developed progressive hemodynamic instability by the second postoperative day, marked by persistent fever, worsening respiratory failure, and rising vasopressor requirements. There were no clinical or radiological evidence of an anastomotic leak or technical surgical failure. Despite maximal intensive care support, his condition escalated to refractory septic shock and multiorgan failure, culminating in death on the sixth postoperative day.

### Parental perspective

The patient’s father reflected deep distress regarding the rapid deterioration of his son’s health. He noted that after the initial discharge from the primary hospital, the symptoms were assumed to be transient discomfort from minor burns. He expressed gratitude for the surgical team’s aggressive efforts at the tertiary center despite the ultimate tragic outcome.

## Discussion

### Epidemiology and corrosive pathophysiology

EPF is a rare but life-threatening communication between the esophagus and pericardium, allowing air, fluid, or food to enter the pericardial space and cause pericardial effusion, pneumopericardium, pericarditis, or, worse, cardiac tamponade [2, 9]. Historically, benign esophageal conditions represent the majority of reported EPF cases, while malignancy and iatrogenic causes have also been described. The reported proportions vary between studies because of differences in case selection and overlapping etiological classifications [10]. In recent years, however, the widespread use of atrial fibrillation ablation and advanced endoscopic procedures has shifted the etiologic profile, with iatrogenic injury now representing the leading cause of EPF, followed by malignancy [11, 6]. The present case is distinctive in that it occurred in a 3-year-old child and resulted from battery acid ingestion, an exceptionally rare cause of EPF in the pediatric population.

Although esophageal perforation is a recognized complication of corrosive ingestion, fistulization more commonly involves the tracheobronchial tree (approximately 3%), whereas extension into the pericardium remains exceedingly rare. In a large study of 1,260 patients with massive corrosive ingestions, the incidence of esophageal fistulization into the mediastinal structures was only 0.2% [12, 13]. Unlike alkalis, which cause liquefactive necrosis and rapid deep penetration, strong acids (such as sulfuric acid found in car batteries) typically cause coagulative necrosis, creating a protective eschar that initially limits transmural injury. However, this protection is temporary; the critical danger arises during the phase of eschar sloughing, which typically occurs 2 to 3 weeks post-ingestion. This separation leaves the thinned esophageal wall vulnerable to delayed perforation and progressive transmural necrosis, leading to delayed clinical deterioration rather than a truly asymptomatic period [3, 4, 12]. The anatomical proximity of the esophagus to the posterior pericardium facilitates the direct extension of this necrotic process into the pericardial sac once the eschar separates [14]. This delayed progression following initially underestimated, non-specific symptoms-rather than a completely symptom-free period-explains the sudden septic collapse observed in our case, which occurred three weeks post-ingestion.

### Diagnostic evaluation

The clinical presentation of EPF is highly variable and depends on the underlying etiology and the size of the fistula. Symptoms may range from non-specific and mild manifestations—such as chest pain, dysphagia, fever, cough, and dyspnea—to severe, life-threatening complications including pericarditis, sepsis, and cardiac tamponade [11, 15]. Owing to the nonspecific nature of these early symptoms and the subacute timeline associated with corrosive acid injury, diagnosis is frequently delayed, with many patients presenting 1–3 weeks after symptom onset. On auscultation, a characteristic “water-wheel” murmur may be detected, reflecting the admixture of air and blood within the pericardial space [6, 13, 16]. In our patient, persistent epigastric pain, progressive dyspnea, dysphagia, recurrent fever, and feeding-related cough reflected this progressive transmural injury and subsequent pericardial involvement.

Diagnosis of EPF requires a high index of clinical suspicion. Chest radiography and transthoracic echocardiography are useful initial investigations and may reveal pneumopericardium or pericardial effusion; however, they are insufficient to confirm the presence of a fistula or delineate its tract [15, 16]. Contrast-enhanced CT is an important diagnostic modality for evaluating suspected esophageal perforation and associated complications. However, diagnosis of EPF often requires a multimodal approach combining CT, contrast esophagography, echocardiography, and endoscopy according to the patient’s clinical condition and available expertise [17]. CT allows accurate identification of the fistula, its anatomical level, and associated findings such as esophageal dilation and inflammatory changes [7]. A water-soluble contrast esophagogram (WSCE) is also a frequently used initial diagnostic test when a fistula is suspected, with a reported sensitivity of 82.8% [11]. Upper endoscopy may provide direct visualization of the defect but carries a risk of air insufflation–related complications, including air embolism and hemodynamic instability, particularly if performed before excluding an esophagoatrial fistula or prior to adequate pericardial drainage [18, 19]. Similarly, in our case, a multimodal diagnostic approach was essential to confirm the diagnosis and guide management.

### Management considerations and surgical strategy

Initial management focuses on prompt pericardial drainage and the administration of broad-spectrum antibiotics with antifungal coverage, given reports of fungal pericarditis in EPF. Definitive management consists of either surgical repair or endoscopic stent closure of the fistula [14, 20–21]. Due to the rarity of EPF, comparative evidence between surgical and endoscopic approaches is limited and mainly derived from case reports and small case series. Therefore, no definitive conclusions regarding survival differences can currently be made. Management should be individualized according to the cause, extent of injury, contamination, and patient stability. Surgical intervention is generally preferred in the presence of abscess formation, ongoing hemorrhage, or friable esophageal tissue, whereas endoscopic stenting may be appropriate for early presentations in hemodynamically stable patients [6, 11, 19]. However, in resource-constrained environments where advanced pediatric endoscopic devices such as pediatric covered stents, EVT, or OTSCs are unavailable, open surgery remains the sole life-saving option. In our patient, delayed presentation, extensive corrosive injury, friable esophageal tissue, and the absence of advanced endoscopic resources necessitated esophagectomy with reconstruction. Despite a technically successful surgery, the overwhelming septic burden resulted in a fatal outcome, underscoring the severity of this condition. Overall prognosis remains poor, particularly in cases related to malignancy, and is largely determined by early diagnosis and timely intervention [11, 22].

The decision to perform an immediate single-stage gastric pull-up reconstruction in a heavily contaminated, septic field remains a complex surgical debate. While staged damage-control surgery (cervical esophagostomy, gastric closure, and feeding jejunostomy) minimizes initial operative duration during acute sepsis, single-stage reconstruction was selected in our case based on six crucial intraoperative and contextual rationale. First, intraoperative exploration revealed a favorable intraoperative environment where, despite the patient’s septic state, there was a complete lack of gross macroscopic contamination (e.g., thick pus or food debris) within the mediastinal and pericardial spaces, with inflammatory fluid being strictly localized and easily cleared with irrigation. Second, the conduit quality was excellent, as direct visual inspection confirmed that the stomach was entirely viable with robust vascular perfusion, having been spared from the corrosive acid. Third, the patient demonstrated notable physiological reserve by maintaining sustained hemodynamic stability throughout the esophagectomy, which prompted the team to opt for a definitive procedure to avoid the physiological “second hit” of a future major reconstruction. Fourth, immediate reconstruction achieved definitive surgical source control through the complete removal of the necrotic esophagus and immediate restoration of gastrointestinal continuity. Fifth, geographical and post-operative care limitations weighed heavily in decision-making; creating a temporary cervical esophagostomy (spit fistula) and feeding jejunostomy would have committed the child to complex, long-term stoma management, which, given the family’s residence in a remote rural area with poor access to specialized healthcare, posed a prohibitively high risk of outpatient morbidity—such as severe stoma-site excoriation, uncontrolled secretions, tube displacement, and subsequent severe malnutrition. Finally, regarding the nature of the clinical outcome, the patient’s post-operative course showed no clinical or radiological evidence of an anastomotic leak or technical failure of the reconstruction. Ultimately, the patient’s demise was driven by the severe inflammatory burden, delayed presentation, and refractory septic shock established prior to surgery rather than a technical failure of the surgical repair.

### Comparative literature analysis

Because reported cases of pediatric EPF resulting specifically from corrosive battery acid ingestion are virtually nonexistent, we conducted a comparative review of published pediatric EPF cases across various etiologies (Table 1). This case analysis illustrates that while non-corrosive pediatric EPF such as foreign body ingestion or iatrogenic trauma can occasionally be managed conservatively or by direct localized repair, severe corrosive ingestion induces extensive, widespread transmural tissue destruction that uniformly requires radical surgical resection.

**Table 1:** Comparative summary of reported pediatric cases of esophagopericardial fistula (EPF)

Case	Age/Sex	Etiology/Caustic Agent	Diagnostic Method	Treatment	Outcome
Muñoz-González S et al. 2024 [23]	16 yrs/ Male	Congenital esophageal atresia with colon interposition	CT, EGD	Endoscopic vacuum-assisted closure therapy with TPN and broad-spectrum antibiotics	Survived
Nasser et al. 2022 [24]	3 yrs/Female	Post-dilation Esophageal Peroration into pericardium	Lateral CXR, WSCE, Serial Echo	Conservative (TPN, gastrostomy suction, broad-spectrum antibiotics, serial Echo)	Survived
Soni et al. 2016 [14]	3 yrs/ Male	Button battery ingestion	CXR, Echo, EGD	Conservatively with antibiotics	Survived
Han et al. 2008 [25]	1 year/ Male	Repeated vomiting	WSCE, CT, EGD	Left thoracotomy with pericardial suturing around the esophageal fistula with a supporting muscle flap	Survived
Mignosa et al. 2005 [26]	15 mths/ Female	Foreign body ingestion	TTE, CXR	Median sternotomy with defect closure by pledget-supported 4 − 0 Prolene suture and placing high-pressure suction drains	Survived
Current Case 2026	3yrs/male	Battery acid ingestion	Echo, WSCE, CT, EGD	Total esophagectomy and gastric pull-up	Died (Septic shock)

Abbreviations: CT: computed tomography; CXR: chest x-ray; EGD: esophagogastroduodenoscopy; TPN: total parenteral nutrition; TTE/Echo: transthoracic echocardiography; WSCE: water-soluble contrast esophagogram

## Conclusion

Esophagopericardial fistula is a rare and potentially lethal complication of corrosive ingestion in pediatric patients. Early recognition, rapid diagnosis, and timely multidisciplinary intervention are critical to improving outcomes. This case highlights the devastating consequences of delayed presentation and emphasizes the additional challenges posed by limited access to minimally invasive therapeutic options in resource-constrained settings. Prompt transfer of pediatric corrosive ingestion cases to specialized tertiary centers is essential to mitigate delayed, catastrophic complications.

## Data Availability

No datasets were generated or analysed during the current study.

## Declarations

### Guarantor

Anas Al-Kubati.

## Abbreviations

EPF: Esophagopericardial fistula
WSCE: Water-soluble contrast esophagogram
EGD: Esophagogastroscopy
CT: Computed tomography
NPO: Nil per os
FC-SEMS: Fully Covered Self-Expanding Metal Stent
EVT: Endoscopic Vacuum Therapy
OTSC: Over-The-Scope Clips
